# Social Cost of Leptospirosis Cases Attributed to the 2011 Disaster Striking Nova Friburgo, Brazil

**DOI:** 10.3390/ijerph110404140

**Published:** 2014-04-15

**Authors:** Carlos Pereira, Martha Barata, Aline Trigo

**Affiliations:** 1 Sergio Arouca National School of Public Health (ENSP), Oswaldo Cruz Foundation (FIOCRUZ), Rua Leopoldo Bulhoes, 1480Manguinhos, Rio de Janeiro 21041-210, Brazil; 2 Oswaldo Cruz Institute (IOC), Oswaldo Cruz Foundation (FIOCRUZ), Avenida Brasil, Manguinhos 4365, Rio de Janeiro 21040-360, Brazil; E-Mail: barata@ioc.fiocruz.br; 3 Celso Suckow da Fonseca Federal Center of Technological Education—CEFET/RJ, Avenida Maracana, 229, sala 322 Maracana Bloco E, Rio de Janeiro 20271-110, Brazil; E-Mail: amonteiro@cefet-rj.br

**Keywords:** disaster assessment, cost of illness, health care costs

## Abstract

The aim of this study was to estimate the social cost of the leptospirosis cases that were attributed to the natural disaster of January 2011 in Nova Friburgo (State of Rio de Janeiro, Brazil) through a partial economic assessment. This study utilized secondary data supplied by the Municipal Health Foundation of Nova Friburgo. Income scenarios based on the national and state minimum wages and on average income of the local population were employed. The total social cost of leptospirosis cases attributed to the 2011 disaster may range between US$21,500 and US$66,000 for the lower income scenario and between US$23,900 and US$100,800 for that of higher income. Empirical therapy represented a total avoided cost of US$14,800, in addition to a reduction in lethality. An estimated 31 deaths were avoided among confirmed cases of the disease, and no deaths resulted from the leptospirosis cases attributed to the natural disaster. There has been a significant post-disaster rise in leptospirosis incidence in the municipality, which illustrates the potential for increased cases—and hence costs—of this illness following natural disasters, which justifies the adoption of preventive measures in environmental health.

## 1. Introduction

When communities struggling with social, environmental, or health vulnerabilities are affected by extreme events such as hard rains, the effects may worsen enough to cause disasters that could lead to a wide range of material, environmental and human loss. These events may eventually lead to an increased occurrence of some diseases [1,2,3], such as leptospirosis, which is often connected to episodes of heavy rainfall. In the State of Rio de Janeiro (Brazil), the uncontrolled urban growth in naturally vulnerable areas, the urban soil compaction, and the deficits in basic sanitation services increase the frequency and severity of environmental disasters caused by extreme events [4]. The State of Rio de Janeiro has some regions that are seriously vulnerable to extreme events, including the Mountain Region that comprises 14 municipalities.

**Figure 1:**
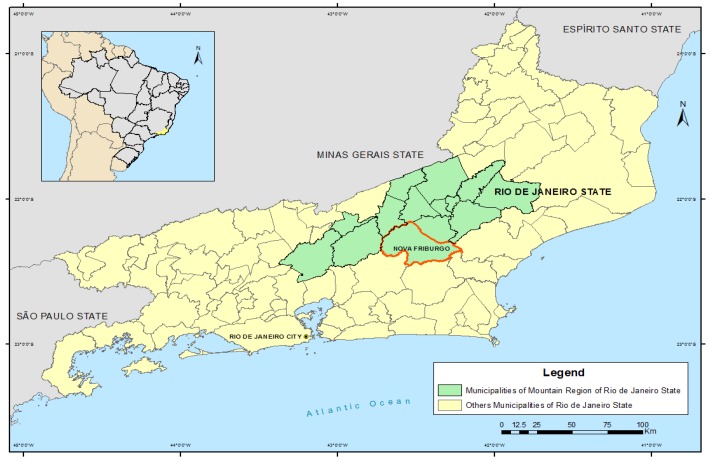
Location of municipalities in the Mountain Region of Rio de Janeiro State (Brazil). Highlight for Nova Friburgo.

Note: Source—Archive of authors, 2012. Produced from the database of the Brazilian Institute of Geography and Statistics (IBGE) [9].

Barata *et al.* [5] demonstrated that the municipalities of Nova Friburgo, Petropolis and Teresopolis, located in the Mountain Region, were among the most susceptible to extreme climate-related events due to their geomorphology and human occupation, with a high potential for human, material and environmental damage [6]. Indeed, on the night of January 11, 2011, heavy rainfall in the Mountain Region caused one of the worst mass movement disasters ever recorded in Brazil [7], ranked by the United Nations as the world’s 8th largest landslide in the last 100 years, as reported by Busch and Amorim [8]. The death toll amounted to more than 900, and another nearly 35,000 people either lost their homes or were displaced in the affected municipalities of the Mountain Region [8], illustrated in Figure 1. 

Nova Friburgo, highlighted in Figure 1, was one of the most severely affected counties, where 3,000 landslides were recorded [8], along with damage to water, power, transport, telecommunications and health services. In this municipality alone, 429 people were killed, and 3,220 were left homeless [10]. The disaster entailed costs to the health sector and society at large, some of which have been ignored, such as the social cost of leptospirosis cases attributed to the calamity.

Leptospirosis was one of the diseases that demonstrated a sharp rise in occurrence after the disaster; in Nova Friburgo, the county was targeted with specific measures of epidemic control and notification. The real cost of these cases to the municipality of Nova Friburgo has not yet been measured. Underestimated costs may hinder the development of both disease control and disaster prevention strategies because factual analysis remains limited to the immediately visible environmental, material, and human damage.

Analysis of the financial and social impact is important for disaster-related diseases, such as leptospirosis, because the results may signal how much an extreme event can increase the cases—and hence the costs—of the illness. Therefore, the aim of this paper is to estimate the social cost of leptospirosis cases attributed to the disaster that struck Nova Friburgo (State of Rio de Janeiro, Brazil) in 2011.

## 2. Experimental Section

This study includes cost-of-illness analyses for confirmed leptospirosis cases, and avoided cost analyses for syndromic measures adopted in the county. The term “social cost” refers to the total costs and includes both society and health care system costs (*i.e*., represents not only a portion of costs allocated to the population, but integrates the sum of all costs of a disease).

### 2.1. Scope

Nova Friburgo keeps records of past health response actions that were adopted, which provided ground for the economic assessment as described in this article. All confirmed cases of leptospirosis attributed to the disaster by the local health authorities were included in this study. These cases had onsets of symptoms between 12 January and 22 March 2011. The recommendations contained in the Guide to Epidemiological Surveillance from Brazil [11] were adopted for confirmation of the cases. These recommendations included the procedures for epidemiological research and conducting laboratorial tests and clinical research for suspected cases.

The laboratory method of choice depended on the developmental stage of the disease in the patient, and the two most used in Brazil are the Enzyme Linked Immunosorbent Assay (ELISA) and Microscopic Agglutination Test (MAT) [11]. The number of laboratory tests performed by each suspected case was variable in Nova Friburgo. The criteria for confirmation were either clinical-laboratory—when the laboratorial test results and the signs and symptoms presented were compatible with the disease—or clinical-epidemiological—when presented symptoms were associated with epidemiological antecedents (such as contact with flood), and for some reason, samples were not available for laboratory tests or a single sample collected before the 7th day of the illness did not have a positive result. In the medical records, there was only information if the case was confirmed or discarded, without information on probable cases.

### 2.2. Source of Data

The study used secondary data supplied by the Health Surveillance sector and the Primary Health Care sector of the Municipal Health Foundation (FMS) of Nova Friburgo. Several of these data are not openly available. Pertinent data were obtained from reports of epidemiological surveillance, financial and management reports, medical charts of leptospirosis notifications, and charts of empirical therapy. Data collection was conducted between December 2012 and April 2013. Furthermore, data on county characterization were obtained from the Brazilian Institute of Geography and Statistics (IBGE) [12], the Brazilian Support Service for Micro and Small Enterprises (SEBRAE) [13], the Federal Ministry of Health [14], and the National Registry of Health Establishments (CNES) [15].

### 2.3. Costs to the Health System

The Unified Health System (SUS) defrays public health services in Brazil, and its expenses are named direct costs, which include medical costs and non-medical costs. The direct medical costs consist of health system expenses, including both inpatient and outpatient procedures. The expenses incurred from patient companion care (*i.e*., follow-up of patients in hospital) represent direct non-medical costs. Data on direct medical costs were gathered from financial and epidemiological surveillance reports, as well as records of leptospirosis notifications. These records are maintained by the Municipal Health Foundation (FMS).

The costs from patient companions were calculated on the basis of the companion per diem values listed in the Management System of Table of Procedures, Drugs, Orthotics, Prosthetics and Special Materials of the SUS (SIGTAP) [16]. Nevertheless, this cost was only included for those cases in which the SUS allows for the permanent companions, specifically for patients under 18 years of age or aged 60 and over [17].

Information about diagnostic tests was retrieved from the medical charts to record the disease. Information on the type and quantity of performed exams was recorded from the patient medical charts, and the costs were accessed from SIGTAP [16] and the Outpatient Information System (SIA) [18]. However, both databases showed zero cost for all the diagnostic tests for leptospirosis. A possible explanation is that specific kits for leptospirosis tests are usually made available to the public health system laboratories, so no additional costs would result from testing. The Central Laboratory Noel Nutels (LACEN-RJ), a major reference to Nova Friburgo for diagnostic tests, was consulted on the matter, and the organization informed that the State Network of Public Health Laboratories typically request the Federal Ministry of Health for the *Leptospira* ELISA kit, containing 96 individual tests. Because no other types of test kits are used by this institution [19], we could only estimate the cost of this particular diagnostic test.

### 2.4. Costs to the Society

Society has to bear some of the costs related to a disease. In this study, the costs to society were represented by productivity loss (PP), which consists of the labor input that the patient failed to apply to their job activities due to absenteeism relative to the disease. This burden is imposed on the employer because the employee still gets paid for the missed work days. This cost was calculated using Equation 1, based on Motta [20]:

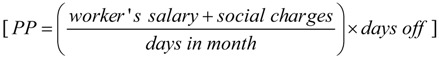
(1)


Because no records on absenteeism in the city were available, productivity loss had to be estimated. The recovery period from leptospirosis may last from one to two months, and urinary elimination of *Leptospiras* may persist for months after symptoms have disappeared [11]. Absenteeism is therefore expected among patients stricken with the disease, due to either the illness recovery period or reasons of sanitary impediment.

Considering that the PP represents the burden imposed on the employer, who pays only for the first 15 days of employee absence, we could exclude those cases in which absence from work was extended for longer periods. The maximum limit for productivity loss was set at 15 missed work days. This number is justified by the analysis of the statistical yearbooks of the Brazilian Ministry of Social Security [21]. The data shows that the monthly average of benefits granted due to leptospirosis under both the Welfare Illness Aid and Accident-related Illness Aid categories in 2011 was low—26.25 benefits per month for the entire country. The data demonstrates that most reported cases of leptospirosis resulted in absences from the workplace for periods not longer than 15 days and are therefore not included in these statistics. The Brazilian social security system only pays benefits from the 16th day of absence.

Two different scenarios were adopted in the calculation of total productivity loss: one refers to the minimum known period of absenteeism, in which lost productivity is equal to the number of hospitalization days; the other refers to the maximum possible period of absenteeism, which occurs if both outpatient and hospital patients are absent for the period most costly to the employer (*i.e*., 15 days). Finally, PP was considered as the range of values set between the parameters calculated for the two scenarios.

The reference wage values adopted in the calculations included the national minimum monthly wage in 2011 [22,23], the lowest minimum monthly wage in the State of Rio de Janeiro in 2011 [24], and the average monthly wage in the municipality of Nova Friburgo [25].

### 2.5. Empirical Therapy and Avoided Cost Analysis

Empirical therapy adopted in Nova Friburgo consisted of the administration of specific medication for leptospirosis treatment even if there was no diagnostic confirmation, a procedure in which the presence of three characteristic symptoms of the disease was required [10]. The major symptoms to define an eligible case for empirical therapy were fever, myalgia and headache, but the other symptoms of the disease were also considered. This measure aimed to prevent worsening of the patients’ condition before their diagnoses were confirmed and avoid increased social costs caused by the disease. The drugs recommended in the Guide to Epidemiological Surveillance were the following: doxycycline (the main drug used in Nova Friburgo), amoxicillin, ampicillin and penicillin. In Brazil, empirical therapy is also called the syndromic approach.

The necessary information for calculating the avoided cost of treatment was obtained from charts used for syndromic surveillance, namely the medications administered, duration of treatment, and dates of procedures. The cost of the syndromic approach comprised drug expenses, as well as the cost of the health care team performing the procedure.

As reported by the Epidemiological Surveillance Service of the municipality, the State of Rio de Janeiro Health Department professionals performed the first procedures and interventions at the calamity site. After leaving, the City staff continued the job. Because there was no accurate information about who actually implemented the measures or how many man-hours were employed in the work, each intervention was attributed the value of US$26.98, which corresponds to 15-minute work by a team consisting of a physician, a nurse, and a community health agent. The calculated value was based on the average wages for these professionals in the region [26,27,28,29]. Drug values were obtained from the Drugs Price List from 2011, which was available on the National Health Surveillance Agency (ANVISA) website [30].

To assess the health sector avoided costs (with not having a local parameter for the pre-disaster period), the ratio of inpatient and outpatient cases in Nova Friburgo was compared to that observed at the national level. These cases were estimated by research conducted about cases of leptospirosis that occurred throughout Brazil during the year 2008 [31], the latest year for which assessment of this type was held in the country. Although this calculation is an estimate of a period other than the period analyzed in this study (2011), it is the most current available parameter. To use this estimate, we assumed that no major changes in the behavior of the disease that could alter this parameter occurred between 2008 and 2011. The number of prevented deaths by empirical therapy was estimated by using the average disease lethality recorded in Nova Friburgo between 2001 and 2010.

### 2.6. Ethical Criteria

This study was approved by the Ethics Committee of the National School of Public Health Sergio Arouca—ENSP/Fiocruz and by the Ethics Committee of the Oswaldo Cruz Institute—IOC/Fiocruz, under CAAE 907112.2.0000.5248. FMS Nova Friburgo signed a Term of Consent for the conduction of the study and collection of secondary data. Additionally, the researchers signed a Confidentiality Agreement.

## 3. Results and Discussion

In Nova Friburgo, approximately 182,000 people live within a 933.4 km^2^ area, which corresponds to a population density of 195 people per km^2^ [12]. This area is one of the three most populous municipalities in the Mountain Region of the State of Rio de Janeiro, concentrating nearly 20% of its entire population [14]. The Human Development Index (HDI) for the municipality is 0.810. The main economic activities are food and beverages services (service sector), clothing and accessories retail (commerce), underwear manufacturing (industrial sector) and cattle raising (agricultural sector). Most households (29%) fall under C1 class, in which the monthly family income is approximately US$889.28 [13].

Of all current health facilities in the city, 65.5% are private; the other establishments (34.5%) belong to the municipal public service [12]. The core public facilities comprise [15]: one Psychosocial Care Center (CAPS), 19 Basic Health Units and Family Health Strategies, one specialized clinic, one Specialized Hospital (Maternity), one General Hospital, three Polyclinics, one Emergency Unit (UPA) and two Terrestrial Mobile Units.

In the post-disaster period between January and March 2011, 525 suspected cases of leptospirosis were treated in public health units, and 177 of these cases received diagnosis confirmation (98 by clinical/laboratory criteria, and 79 by clinical/epidemiological criteria). According to information from the Epidemiological Surveillance Service, the disaster caused an environmental imbalance in Nova Friburgo that changed the leptospirosis behavior previously observed in the county. Therefore, all cases recorded until March 2011 were attributed to the disaster. Indeed, there was an atypical number of confirmed cases in the county in 2011, as shown in Table 1, which displays information obtained from the Brazilian Case Registry Database (SINAN) [32]. This table contains only reported cases with a confirmed diagnosis.

**Table 1:** Confirmed cases of leptospirosis in Nova Friburgo between 2001 and 2011.

Year	2001	2002	2003	2004	2005	2006	2007	2008	2009	2010	2011 *
Confirmed cases	3	1	2	2	4	0	9	5	6	7	177

Notes: Source: Brazilian Case Registry Database (SINAN) [32]. * Data supplied by Municipal Health Foundation (FMS) of Nova Friburgo. 2011 data in the SINAN still subject to review.

Descriptive statistics of outpatient cases are presented in Table 2, and those of hospitalized cases in Table 3. According to the data from these two tables, the majority of cases of leptospirosis attributed to the Nova Friburgo disaster were addressed in outpatient care, and most patients were male. The mean and median ages of hospitalized patients were higher than those of patients receiving outpatient care. Also based on the two tables, the most performed diagnostic test among both hospital and outpatient cases was the Enzyme Linked Immunosorbent Assay (ELISA), which can offer faster results than the Microscopic Agglutination Test (MAT) test, currently the gold standard for leptospirosis diagnosis [33,34]. Among the other tests applied were: indirect immunofluorescence test for leptospirosis and tests for hepatitis A and C.

Most of the cases were cured, although the data on the disease progression of two patients is unavailable. The City Epidemiological Surveillance Service confirmed that no deaths due to leptospirosis were recorded among the cases assigned to disaster. The costs associated with confirmed cases are presented below.

### 3.1. Direct Costs to the Health Sector

The costs to the health system reported in this section include outpatient and hospital expenses. The cost of the 149 confirmed cases treated in outpatient care was nearly US$1,500 in total. This amount includes costs for diagnostic tests and specialist medical consultations expenses. The median outpatient cost was US$9.79. For the 28 confirmed cases that required hospital care, the total cost was nearly US$10,700. The total hospital care cost includes the costs of diagnostic tests, professional services, hospital services and companion care per diems. The median hospital cost was US$346.44. In summary, the 177 confirmed cases of leptospirosis cost US$12,200 in expenses by the Health Care System, which was considered as direct costs by the Department of Health.

**Table 2:** Descriptive statistics of outpatient leptospirosis cases in Nova Friburgo.

Variables	Valid N (%)	Years
Total number of confirmed cases	149	
Gender	149	
Male	87 (58.39)	
Female	62 (41.61)	
Age	148	
Mean		33.01
Median		31.00
Standard deviation		14.01
Diagnostic tests performed	159	
Elisa	133 (83.65)	
Mat	8 (5.03)	
Other	18 (11.32)	
Criteria for classification	149	
Confirmed by clinical/laboratory criteria	74 (49.66)	
Confirmed by clinical/epidemiological criteria	75 (50.34)	
Evolution	148	
Cure	148 (100.00)	
Death due to leptospirosis	0 (0.00)	
Death due to other causes	0 (0.00)	

The median costs of leptospirosis were higher than the costs of dengue fever, another disease strongly associated with the Nova Friburgo disaster. Among the confirmed dengue fever cases also related to the 2011 disaster, the median hospital cost was US$274.18, and the median outpatient cost was US$6.35 (data calculated based on statistics provided by the FMS-Nova Friburgo).

### 3.2. Costs to Society

Together, the 28 leptospirosis patients who were hospitalized stayed for 179 days in health facilities. Of these cases, 26 patients were of working age and were absent from work for 173 days, which corresponds to a PP of US$3,300 based on the national minimum wage of 2011 [22,23]. This value reaches US$3,700 when the lowest minimum wage in the State of Rio de Janeiro in 2011 is considered [24], and US$5,700 when calculated according to the 2010 average wage in Nova Friburgo [25].

Among the 149 cases treated in outpatient clinics, 129 patients were at an economically active age. The estimated loss for this group ranged from 129 to 1,935 workdays. The estimated PP for outpatient cases ranged from US$2,700 to US$39,800 based on the national minimum wage in 2011 [22,23]; US$3,000 to US$44,800 when considering the lowest minimum wage in the State of Rio de Janeiro in 2011 [24]; and US$4,600 to US$68,700 if using the employee’s average wage of Nova Friburgo [25].

**Table 3:** Descriptive statistics of hospitalized cases with leptospirosis in Nova Friburgo.

Variables	Valid N (%)	Years
Total number of confirmed cases	28	
Gender	28	
Male	18 (64.29)	
Female	10 (35.71)	
Age	28	
Mean		40.93
Median		43.50
Standard deviation		15.94
Diagnostic tests performed	49	
Elisa	41 (83.67)	
Mat	8 (16.33)	
Other	0 (0.00)	
Criteria for classification	28	
Confirmed by clinical/laboratory criteria	24 (85.71)	
Confirmed by clinical/epidemiological criteria	4 (14.29)	
Evolution	27	
Cure	26 (96.30)	
Death due to leptospirosis	0 (0.00)	
Death due to other causes	1 (3.70)	

The total loss of productivity, considering both hospital and outpatient cases, was estimated for two different scenarios: the first assumed only the minimum known amount of missed work days, which is equal to the total days of hospitalization; and the second considered the maximum possible loss, which would result from patients being absent from work for as long as possible (*i.e*., 15 days according to the adopted criteria). Based on the 2011 national minimum wage [22,23], the total PP ranged from US$3,300 to US$47,800. Using the lowest minimum wage in the State of Rio de Janeiro in 2011 [24], the total PP varied between US$3,700 and US$53,800. Total PP reached values between US$5,700 and US$82,600 based on average worker wages in Nova Friburgo [25].

### 3.3. Empirical Therapy

Empirical Therapy charts were recovered for 157 (88.70%) of the 177 confirmed cases. The remaining 20 cases (11.30%) had no information. For cases that used the syndromic approach, costs were estimated to be US$4,400 for the health care team that applied the measures and US$1,600 for pharmaceutical expenditures. In total, the estimated cost of the syndromic approach in the county was US$6,000. The cost of the syndromic approach was absorbed by the local health service. The median cost of the syndromic approach among confirmed outpatient cases was US$26.98 and US$46.35 for hospital-confirmed cases.

### 3.4. Avoided Cost Analysis

Empirical Therapy substantially reduced the severity of cases and avoided deaths due to leptospirosis. In Nova Friburgo, only 28 of the 177 confirmed cases required hospitalization. Moreover, there were no deaths among the leptospirosis cases that were attributed to the disaster.

Among the confirmed cases of leptospirosis in the county, the treatment ratio was one hospital case to 5.32 outpatient cases. Based on the estimated ratio for episodes that occurred throughout Brazil during 2008 [31]—0.90 outpatient cases to each hospital case—and considering no major changes in the behavior of disease occurred until 2011, the analysis of avoided cost was based on this national estimate. 

Assuming that the empirical therapy (syndromic approach) reduced the ratio of hospital cases in the county and Nova Friburgo would present the same rates assessed in the 2008 national research data, the number of confirmed hospital cases would be 93 (instead of 28), and 84 outpatient cases (instead of 149) would have been recorded. This calculation would result in a total of 177 cases, which was the number of confirmed cases actually recorded. If so, the confirmed cases alone would have cost US$33,000 to the health system. In reality, this cost was US$18,200 (treatment costs and empirical therapy). Therefore, under these circumstances, the syndromic approach avoided US$14,800 in costs to the Health System.

The syndromic approach may have also reduced the costs to society. Between 2001 and 2010, seven deaths due to leptospirosis in Nova Friburgo were reported to the Mortality Information System (SIM) [35]. Considering the total number of leptospirosis cases in the municipality reported in SINAN [32] for the same period, the average lethality of the disease equals 17.95%. In 2011, when empirical therapy was applied, no deaths due to leptospirosis attributed to the natural disaster were recorded. If this reduction in mortality is assumed to be a result of the syndromic approach, then 31 deaths were avoided among the 177 confirmed cases of the disease.

### 3.5. Total Cost of Illness

The total costs incurred by the health system, arising from professional services, outpatient and hospital services, diagnostic tests and empirical therapy, was US$18,200. Because the costs to society, represented by lost productivity, varied according to each scenario—from US$3,300 to US$47,800 in the lowest wage scenario, from US$3,700 to US$53,800 in the intermediate wage, and US$5,700 to US$82,600 in the highest wage scenario—the total social cost of leptospirosis cases in Nova Friburgo due to the disaster of January 2011 may have had various different values: from US$21,500 to US$66,000 in the scenario displaying less productivity loss; from US$21,900 to US$72,000 in the intermediate productivity loss scenario; and US$23,900 to US$100,800 in the larger productivity loss scenario. Table 4 contains a summary of the variables assessed in hospital and outpatient cases in Nova Friburgo that were attributed to the 2011 natural disaster.

Previous studies have evaluated the cost of leptospirosis in other settings. Souza *et al.* [36] estimated the costs of hospitalization and years of potential life lost associated with leptospirosis in Brazil in 2007. The study found that 331 patients died, corresponding to an average of 30 years of potential life lost for each death. The costs of hospitalization were approximately US$440,000 relating to 817 hospitalized cases. Nearly US$54,500 in productivity loss occurred in 707 economically active cases.

**Table 4:** Summary of costs associated with confirmed leptospirosis cases attributed to the 2011 disaster in Nova Friburgo.

Variables	Hospital	Outpatient
**Confirmed cases**	28	149
**Variables related to care**		
Average length of stay (days)	6.36	-
Total cost (US$)	10,736.00	1,488.97
Mean cost (US$)	383.43	9.99
Median cost (US$)	346.44	9.79
**Empirical therapy**		
Valid N	22	135
Total cost (US$)	921.86	5,093.01
Mean cost (US$)	41.90	37.72
Median cost (US$)	46.35	26.98
**Loss of productivity**		
Economically active N	26	129
Loss based on the median income of the county (US$) *^,^’	5,754.08	4,581.95–68,729.31
Loss based on the state minimum wage (US$) *^,^”	3,753.15	2,988.62–44,829.37
Loss based on national minimum wage (US$) *^,^^#^	3,337.29	2,655.28–39,829.13

Notes: * Off work period ranging from one to 15 days for outpatient cases. ’ Average income of the local population in the amount of US$591.98 [25]. ” It was considered the lowest value of 2011, equivalent to US$386.13 [24]. ^#^ Value between January and February 2011: US$343.01 [21]. Value effective as of March 2011: US$346.19 [23].

Because no deaths occurred in Nova Friburgo, there were no lost years of potential life in the cases of leptospirosis associated to the disaster. The average hospitalization cost was higher in the study by Souza *et al.* (US$538.55) whereas in Nova Friburgo, the average cost was US$383.43. The average loss of productivity was also greater in the study by Souza *et al.* (US$770.86) whereas in this study, the highest average was US$221.31. In Brazil, not all cases are related to disasters, and the search does not always enough active to start the treatment in the early stage of the disease. Most cases in Brazil are moderate or severe, with underreporting of cases in the early stage [11]. Because Souza *et al.* intended to study the cases that died, the costs of their study may have been higher because these cases may have been the most serious and complex, required longer treatment, and demanded more health services, which would generate a higher cost for both the health care system and society.

Another study about leptospirosis was performed in New Caledonia [37] where an epidemic of human leptospirosis after heavy rainfalls and floods occurred in 2008. The disaster resulted in 135 cases of leptospirosis and five deaths. Eighty eight patients were hospitalized, generating a direct medical cost of 622,894 € (US$989,100). The remaining 47 patients not hospitalized generated a cost of 14,486 € (US$23,000). The total number of lost workdays was estimated to be 1,431 days, corresponding to a loss of 86,340 € (US$137,100). Altogether, the total cost for the 135 cases was estimated to be 723,720 € (US$1,149,200). The average cost of hospitalization was also higher for the New Caledonia study (US$11,239) compared with this study. The average cost of non-hospital treatment was also higher (US$489). The New Caledonia study presented a single value for the loss of productivity, perhaps by having tracked the cases and obtained more accurate responses. Despite the uncertainty in this estimate for the Nova Friburgo study, it surpassed the New Caledonia loss of productivity (when highest wage scenario is considered). Despite the differences in the estimates, both of these studies illustrate the effect of extreme rains on increasing leptospirosis cases and the cost of illness.

In their review of recent literature on the causes, consequences and responses to natural disasters, Freitas and Ximenes [38] emphasized that leptospirosis has had a major effect on health, especially in flooding episodes. In fact, in Nova Friburgo, an average of 3.9 confirmed leptospirosis cases per year were reported to SINAN between 2001 and 2010, whereas in the first three months after the disaster, 177 cases of the disease were confirmed, which was 45.38 times the amount of cases as the municipality’s average. Climate scenarios that have been developed for Brazil indicate a probable increase in the number of extreme hydro-meteorological events in the coming years, especially in the South and Southeast regions [39].

Because climate is considered to be a determining factor for the occurrence of infectious diseases such as leptospirosis, which is sensitive to climate variability and extreme events such as floods [40], a higher frequency of cases in these areas can be expected. Despite this connection of leptospirosis with floods, there are other factors that may precipitate its emergence, even during dry seasons. A study developed in Aracaju (State of Sergipe, Brazil) on leptospirosis cases during 2001 to 2007 provided a clear illustration of this situation: the authors found no correlation between rainfall and cases of the illness in the city, concluding that occurrence may increase with rainfall, but even in the absence of rain, a certain amount of cases of the disease will occur [41]. 

Fensterseifer [42] explained the effects of environmental injustice and social inequalities in increase the number of vulnerable groups to the negative effects of environmental degradation and disaster. In the specific case of the health in Nova Friburgo, poorer social groups were not more affected by the disaster than wealthier groups. Many leptospirosis cases were observed in the municipality’s main district and in places that actually represent high economic activity and income concentration levels. The very center of the city, which was heavily impacted, is a good example. Perhaps inequalities have surfaced in access to emergency and response services because those places distant from the urban center and with rugged topography were more difficult to reach, due to barriers in access routes.

Fensterseifer also discussed the extent to which the government should be responsible for damage caused by extreme events. He stressed that the State must seek ways to compensate those affected and meet their basic rights, especially if their vulnerability results from state failure to prevent damage due to climate change. In most cases, the poorest segment of the population comprises the people most affected, who have low autonomy and capacity to respond to the impacts of a disaster [42]. Therefore, the state must act on the resilience of these people, preventing vulnerability and risk situations when everyday life activities resume.

However, aid measures implemented by the State may not adequately incorporate the costs to society caused by the disaster because economic assessments are not routinely performed to support public action. In Nova Friburgo, the estimated cost to society of leptospirosis ranged between US$3,300 and US$82,600, without considering other possible social impacts, such as material losses. Adopting economic assessments of social costs could lead to more coherent compensation, consistent with the magnitude of losses.

The Health Sector expenses with the empirical therapy may have avoided costs to society because no deaths were recorded. External costs were internalized by the health sector through the syndromic approach. In this approach, drugs prescribed by the Federal Ministry of Health [11] for the treatment of leptospirosis were used, including doxycycline, which is recommended for treatment of the early phase of the disease.

A cost-effectiveness analysis [43] of five different strategies for treating mild febrile illness in patients hospitalized with suspected leptospirosis was performed using a hypothetical cohort of patients over 14 years of age, based on another clinical study conducted by the same authors. The strategies analyzed were: (a) non-performance of diagnostic test or use of antibiotic treatment, (b) empiric treatment with doxycycline, (c) use of doxycycline in cases confirmed through the lateral flow test, (d) use of doxycycline in confirmed cases through the MCAT test, and (e) use of doxycycline in cases confirmed by latex test. The empirical treatment had the lowest direct cost and higher effectiveness compared with the other four strategies. As effectiveness was measured by productivity loss by considering the number of days of hospitalization, the empirical treatment resulted in shorter stays for the patients [43]. The empirical treatment described in this study is similar to the syndromic approach adopted in Nova Friburgo, and it corroborates the potential of such measures to avoid costs to society and to health sector as a preventive measure.

Extreme events are recurrent in Nova Friburgo, some of which result in both human and material damage. As reported by Freitas *et al.* [44], flash floods hit Nova Friburgo, Teresopolis and Petropolis in 1988, resulting in 227 deaths and leaving 2,000 homeless. In 2000, floods in the same cities led to five deaths. In 2007, according Barata *et al.* [5], heavy rainfall in Nova Friburgo, Sumidouro, Petropolis and Teresopolis resulted in 23 deaths, 11 of which occurred in Nova Friburgo. Barata *et al.* [5] also reported that flash flooding in 2005 caused one death in Nova Friburgo. This history, coupled with the impact from the 2011 disaster, justifies the adoption of preventive measures because extreme events have sharply increased the incidence of leptospirosis in the municipality.

The issue of the Service of Epidemiological Surveillance needs to be addressed because the success of such measures depends on how their team is strengthened and capable to play their role. The knowledge of the municipality’s epidemiological profile will allow the assessment of existing health risks and provide guidance on what action must be taken to avoid negative impacts to the population. Fostering an epidemiological surveillance system that is sufficiently trained and organized to assess the population’s risks and vulnerability, and also capable of quick response, offering documents and records that will guide the work in the region, is paramount [45]. In Nova Friburgo, despite the team’s strong effort to record activities and a systematized record of all actions, a lack of support and engagement to structure and strengthen this sector is lacking. This support is vital to good health sector performance in disaster management. Undeniably, the work conducted by the team—at the time of disaster comprising five members, currently only four—was successful. Still, management culture needs to emphasize the importance of systematic work in prevention and active work in disaster response. This goal may be developed by the Epidemiological Surveillance.

Leptospirosis is a disease that can be prevented by improved urban and sanitation conditions, and although natural hazards cannot be avoided, the vulnerability of the population can be reduced. Leptospirosis cases related to disasters exert a negative social impact that can be avoided, as well as the economic burden imposed on the health sector and to society in general. This value represents the price that is paid for not spending money on measures of disaster prevention, risk management, urban planning, and vector control.

## 4. Conclusions

There were 177 confirmed cases of leptospirosis in Nova Friburgo that were attributed to the 2011 disaster. The total social cost of these cases ranged between US$21,500 and US$66,000 in the scenario of lower productivity loss, until between US$23,900 and US$100,800 in the scenario with higher productivity loss. The syndromic approach represented a total avoided cost of US$14,800, in addition to a reduction in lethality. These measures proved to be good preventive strategies against the worsening of cases in the municipality, and they also represented savings to the health sector and society. There has been a significant post-disaster rise in leptospirosis incidence in the city, which illustrates the potential for increased cases—and hence costs—of this illness following natural disasters.

It is important to know the full extent of the social costs of health-related outcomes that can be attributed to disasters, such as increased cases of leptospirosis, because only considering the costs of treating the disease can lead to an underestimation of the true impact of these events. The measurement of the economic and social burden of a disease is a useful tool for health and environment management to decide where and how to apply their resources. Furthermore, assessments of the social cost of disasters can subsidize the state on providing aid measures to affected populations.

The authors hope this work will encourage research on the impacts of leptospirosis and social cost of disasters, especially in Brazil, that will engage in practical studies that can be applied in everyday public management of environmental health.
